# Fabrication of practical deformable displays: advances and challenges

**DOI:** 10.1038/s41377-023-01089-3

**Published:** 2023-03-04

**Authors:** Dong Wook Kim, Seong Won Kim, Gyujeong Lee, Jangyeol Yoon, Sangwoo Kim, Jong-Ho Hong, Sung-Chan Jo, Unyong Jeong

**Affiliations:** 1 Department of Materials Science and Engineering, Pohang University of Science and Technology (POSTECH), 77 Cheongam-ro, Nam-gu, 37673 Pohang, Gyeongbuk Republic of Korea; 2 Physical Intelligence Department, Max Planck Institute for Intelligent Systems, Hisenbergstr. 3, 70569 Stuttgart, Germany; 3 Advanced Research Team, Samsung Display Corporation, 1 Samsung-ro, Yongin-si, Gyeonggi-do Republic of Korea

**Keywords:** Displays, Optoelectronic devices and components

## Abstract

Display form factors such as size and shape have been conventionally determined in consideration of usability and portability. The recent trends requiring wearability and convergence of various smart devices demand innovations in display form factors to realize deformability and large screens. Expandable displays that are foldable, multi-foldable, slidable, or rollable have been commercialized or on the edge of product launches. Beyond such two-dimensional (2D) expansion of displays, efforts have been made to develop three dimensional (3D) free-form displays that can be stretched and crumpled for use in realistic tactile sensation, artificial skin for robots, and on-skin or implantable displays. This review article analyzes the current state of the 2D and 3D deformable displays and discusses the technological challenges to be achieved for industrial commercialization.

## Abstract (web summary)

This review article analyzes the current state of the 2D and 3D deformable displays and discusses the technological challenges to be achieved for practical uses and industrial commercialization.

## Graphical abstract

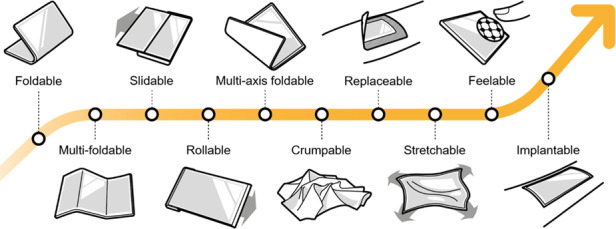

## Introduction

Dailyization of digital is an obvious trend in the electronics industry. Portability, user-convenience, and multi-functionality in daily life are the important issues in the trend. When it comes to portability, displays have made remarkable transition over the past decade from the flat panel liquid crystal displays (LCDs)^1^ to the thin bezel-free display^2,3^. Displays are now evolving to have new form factors, with the advent of the self-luminous displays made of organic light emitting diodes (OLEDs)^4^, quantum dots LEDs (QLEDs)^5^, and micro LEDs (µLEDs)^6^. To keep the pace with the demands for user-convenience and multifunctionality, displays have integrated numerous sensors to feedback various information and have become an indispensable part in a hyper-connected society^7^. The display industry is establishing a technology roadmap that calls for innovative progress in the form factor and functionality^8^.

**Fig. 1:**
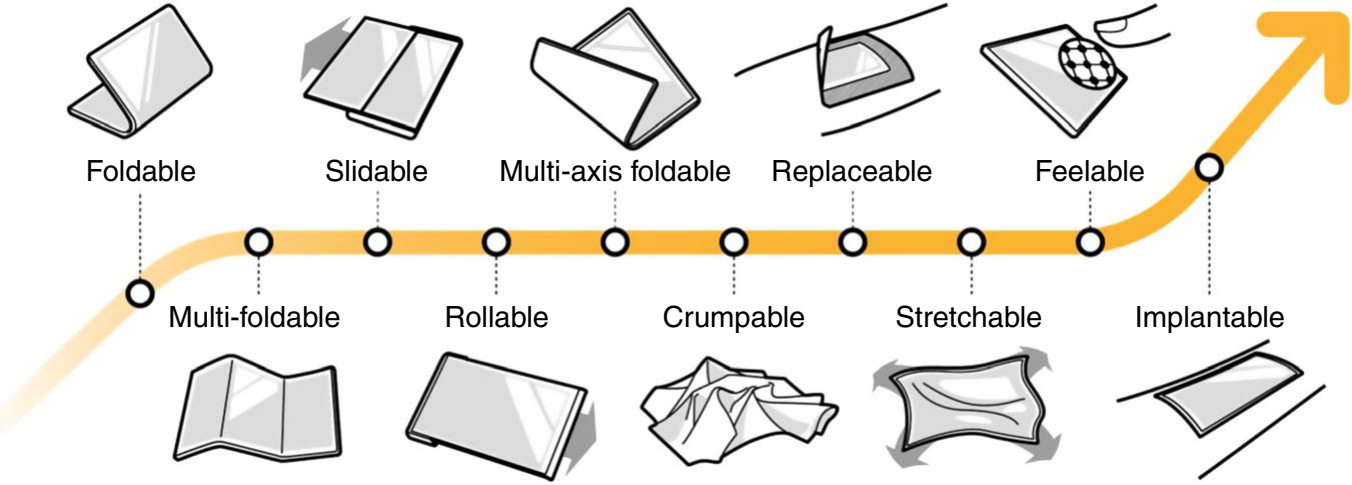
Technological roadmap of deformable displays. It shows the form factor and functional innovation for deformable displays

The display industry has carved out a niche market for deformable displays by successfully demonstrating and commercializing expandable displays^9–12^. Deformable displays are no longer conceptual gadgets watched in science fiction movies. Highly expandable displays can be accomplished by making the screen foldable, multi-foldable, slidable, or rollable in uniaxial direction, and they are expected to be launched to the market in the near future (Fig. 1)^10^. The displays can be further enlarged if they can be folded in multiple axis as observed in a thin sheet of paper. These displays are based on folding and bending in pre-determined directions of two-dimensional (2D) screens fabricated on flexible polyimide substrates^11,12^.

The next direction in the display industry is to secure free-form displays that are deformable in three-dimension (3D)^13–18^. These displays should be manufactured on soft or elastic substrates. The crumpable displays will allow folding without pre-determined folding directions so that they can be crumpled without traces of folding lines^13,14^. The stretchable displays that can be deformed like human skin will be used as artificial skin for robots^15,16^. The electronic skin (E-skin) displays are expected to be replaceable to fit the shape and purpose of the robots^17,18^. Commercialization of stretchable displays has to address several unconventional technological challenges, but these innovative displays can provide more freedom in the dimension and shape of displays as well as unconventional functions^15–18^. The feelable displays with delicate mechanical electrical actuation can deliver tactile sensation in addition to vision and hearing^19,20^. The ultimate direction for portability and convenience is the under-skin implantable displays, for which ethical issues should be discussed^21^.

**Fig. 2:**
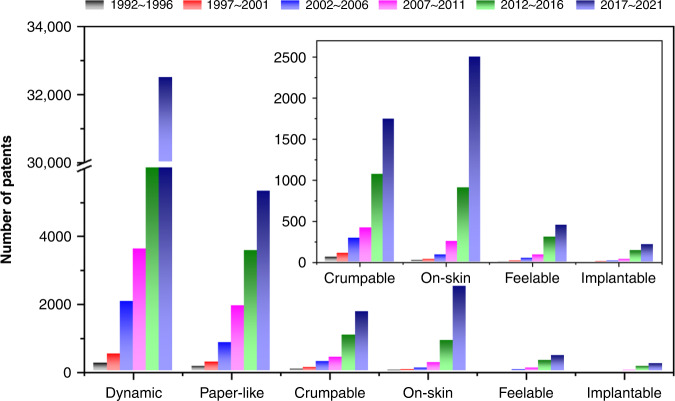
Patent registration for deformable displays. Changes in the number of patents related to deformable displays registered in four countries (United States, China, South Korea, and Japan) every 5 years

Figure 2 shows the changes in the number of patents registered every 5 years in four leading countries (United States, China, South Korea, and Japan) in the deformable displays. The search keywords and screening operators are found in the Supplementary Information (Table S1). We classified the deformable displays into 2D expandable displays and 3D free-form displays. The 2D expandable displays include the dynamic displays (foldable, multi-foldable, slidable, rollable) and the paper-like displays (multi-axis foldable, ultrathin), and the 3D free-form displays include the crumpable, on-skin (replaceable, stretchable), feelable, and implantable displays. The patents for the dynamic displays and the paper-like displays were registered from 1992 and the number increased rapidly from 2000. The number of registered patents for the dynamic displays has continued to increase with the growing market of the foldable mobile phones and reached 32,000 during 2017 − 2021. Since similar concepts and technologies are also essential in the rollable, slidable, multi-foldable displays, the number of patents is expected to further increase. However, the number of registered patents for the paper-like displays increased gradually, reaching about 5,300 during 2017 − 2021. The relatively gradual growth is partially due to the conceptual immaturity of strain engineering and also due to the lack of film-type device components, especially camera and battery. The number of patents for the 3D free-form displays has been growing rapidly since 2012. The patent growths of the textile and on-skin displays are outstanding with the advance of the wearable and healthcare devices, exceeding 1,700 during 2017 − 2021. The number of patents for the feelable displays starts to increase slowly as virtual reality and metaverse emerge recently. The implantable displays are in a conceptual stage so the registered patents are mostly related to optogenetic therapy rather than delivering visual information.

**Fig. 3:**
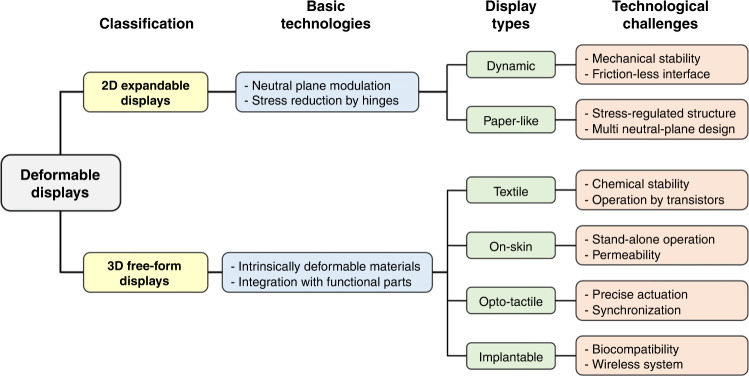
Technological classfication of deformable displays. Basic technologies and technological challenges in the 2D expandable and 3D free-form deformable displays

The basic technologies and further technological challenges in the 2D expandable and 3D free-form displays are summarized in Fig. 3. The 2D expandable displays focus on dynamic shape change by folding and bending. They are based on the neutral plane modulation and hinge design to minimize the stress of the component layers made of conventional rigid materials^22^. Securing mechanical and electrical stability under repeated folding and rolling through frictionless interface design is a major challenge in manufacturing dynamic displays. The paper-like displays must endure harsh mechanical stress that cannot be resolved by a single neutral plane, hence stress-regulated substrates and multilayer interface modulation should be developed^23^. The 3D free-form displays are based on intrinsically deformable materials and novel integration strategies to relieve the applied stress^24^. Although fabrics are ideal substrates for crumple displays, chemical inertness, and reliable device integration are great technological challenges^25^. The replaceable artificial skin displays should acquire simultaneously a high degree of stretchability and mechanical toughness^26^. The opto-tactile displays have the challenges of precise actuation in the millimeter-scale and synchronized operation of visual/tactile information to provide vivid feeling with vision^27^. The on-skin displays should allow permeation of air and moisture to prevent skin irritation in addition to the high stretchability^28^. The implanted displays need long-term biocompatibility and wireless power charging.

There have been several academic review articles on deformable displays^29,30^. This perspective article, focusing on the practical issues, introduces the status of the underlying technologies and discusses the technological challenges for the commercialization of the 2D expandable displays (Section 2) and 3D free-form displays (Section 3). The last section (Section 4) looks at the prospects and assessments of the deformable displays as a guide for future research directions.

## 2D expandable displays

The mobile phone maker companies commercialized foldable mobile phones and the proto-types of rollable display and multi-foldable display have been demonstrated in the display shows and conferences^31,32^. Those deformable mobile phones and tablets can provide a larger screen area in the same device size, giving specialized customer experiences such as multi-tasking, hands-free video conference, and unique selfie pictures.

**Fig. 4:**
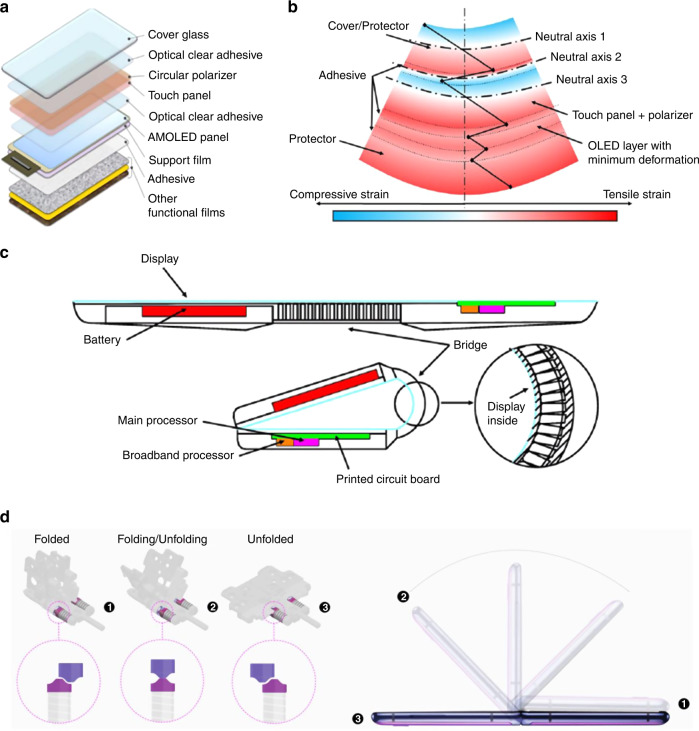
Display structure of the display and hinge adopted in foldable displays. **a** Unit device layers in the foldable displays. Optimization of each layer and design of neutral planes determine the stress distribution applied to cover glass, polarizer, touch screen, and display panel layers. Reproduced with permission^33^ Copyright 2019 Wiley-VCH. **b** Thickness change effect of the optical clear adhesive (OCA) layers on the formation of the neutral planes and the prevention of display delamination. Reproduced with permission^37^ Copyright 2021 ASME. **c** A hinge system adopted in foldable displays to reduce the stress applied to the panel during repeated folding and un-folding cycles. Reproduced with permission^37^ Copyright 2021 ASME. **d** Hideaway dual cam mechanism applied in Samsung Galaxy Z Flip^39^. During folding and unfolding, two cam pieces slide up and down against each other, and the friction between them allows the device to stand alone at different angles Reproduced with permission^39^ Copyright 2020 Samsung Electronics Co., Ltd

The contemporary foldable/slidable/rollable displays are distinguished from the typical flexible displays in two points: engineering the neutral planes and the hinge system under the panel. The foldable display consists of multiple layers in the following order from the bottom (Fig. 4a): optically clear adhesive (OCA), display panel (thin film transistors and light emitting diodes), OCA, polarizer and/or touch sensor, OCA, and cover-window^33^. The brittle layers such as cover window, touch sensor, color filter, and thin film encapsulation layer should be located in the neutral plane to prevent crack formation under folding. The touch sensor and polarizer are integrated into a thin OLED display panel and the ultrathin cover window glass is used to sustain high stress applied to display^34^. Optimization of thickness and stacking order of the OCA and the film layers are critical in designing the neutral planes. The thickness of the OCA is relatively easy to adjust without affecting display performance. A thick OCA layer can absorb most of the strain applied to the display, thereby multiple neutral planes can be designed (Fig. 4b)^33,35–37^. Meng and coworkers published simulation data suggesting that a thicker OCA placed below the cover window can effectively decrease the strain, leading to a reduced risk of delamination^36^. Using a series of hinges is another way of reducing the stress applied to the panel during folding and un-folding process^38^. The hinge is located where the folding occurs, and it helps to move the display synchronously, distributing the stress equally to the folding area of the panel and maintaining flatness when the electronic device is unfolded (Fig. 4c). The hinge creates many user scenarios. The key factors of the hinge system are mechanical stability, precise control of the folding process, stress distribution, and compact size. The detailed design of hinge depends on the manufacturing companies. For instance, the hideaway dual cam mechanism, applied in Samsung Galaxy Z Flip, consists of two cam detents on both ends of the hinge (Fig. 4d)^39^. The cam is a rotating piece of a mechanical linkage that is used to convert rotational motions into linear motions. Two ridged pieces slide up and down against each other, and the friction allows the device to stand alone at different angles. In the fully folded state, the spring continues to push the cam while maintaining a stable closed state. When the device unfolds, the upper cam begins to rise along the ridge of the lower cam and the friction between the flat surfaces of the two cams holds itself open. The device is fully unfolded as the upper cam slides down the slope opposite the lower cam beyond the level of engagement of the flat surface. The spring continues to generate tension through the force that pushes the cam, allowing the device to firmly maintain the unfolded state.

**Fig. 5:**
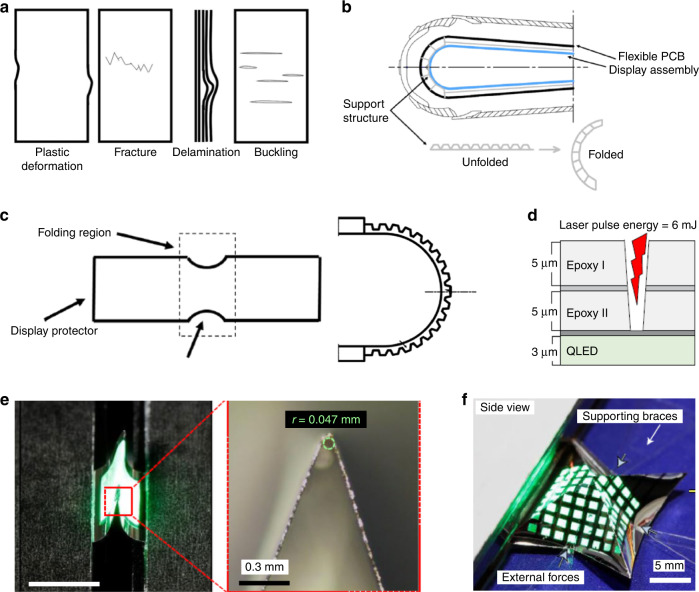
Stress minimization in the 2D expandable displays. **a** Mechanical failure that can occur during repeated folding cycles. Reproduced with permission^37^ Copyright 2021 ASME. **b**, **c** Patterned supporting structure (**b**) and protecting cover layer (**c**) to absorb stress in the folded region. Reproduced with permission^37^ Copyright 2021 ASME. **d** Partial laser etching in the folded region of the top panel layer to realize multi-axis foldable displays. Reproduced with permission^44^ Copyright 2021 Springer Nature. **e**, **f** Folding/bending characteristic of a patterned QLED (**e**) and 3D multi-axis folding of QLED (**f**). Reproduced with permission^44^ Copyright 2021 Springer Nature

The main failure causes of the foldable displays are plastic deformation, fracture, delamination, and buckling as shown in Fig. 5a^37^. The delamination and buckling mainly take place during compression, whereas the plastic deformation happens during extension. The delamination and fracture result in damage of foldable display panel. The creased effect causes distortion of the display in the folding area. A variety of research articles and patents have been published to minimize the stress applied to the folding region. The hinge system for uniform stress distribution in the entire panel is one successful approach. Some patents also propose inserting a patterned soft polymer supporting layer in the folding region to absorb the stress (Fig. 5b)^37,40^. Another is patterning the cover layer (groove, notch design) to minimize the shear stress in the OCA layer and the display layer (Fig. 5c)^37,41,42^. The reduced thickness in the folding region has also been adopted to decrease the stress^43^. The touch sensor and the polarizer (color filter) are integrated into the display and the ultra-thin cover glass has been applied.

The above-mentioned approaches are limited to one-axis foldable display. The architecture of the foldable display panel has been merely unchanged from that of the flexible display panel. To realize the multi-axis foldable displays, a new architecture of the display panel should be designed to accommodate enhanced flexibility. Partial laser cutting of the display panel and the etch-stop layer may achieve 3D deformation of the foldable display, as illustrated in Fig. 5d^44^. The QLED is fabricated on an ultrathin substrate, and the etch stop layers of Ag and epoxy overcoating layers are placed on the QLED. Those layers absorb thermal and ablation damages during laser cutting. With the double etch-stop layers, the neutral plane is located close to the electrode of QLED. The partially cut QLED can be fabricated by optimizing the laser power. The etched device has a smaller bending radius of 0.047 mm when bent (Fig. 5e). The pre-patterned QLED improved the degree of freedom in the folding form factor by multi-directional folding (Fig. 5f). The optimization of the pre-patterning process in terms of accuracy and resolution needs to be developed for practical displays.

## 3D free-form displays

**Fig. 6:**
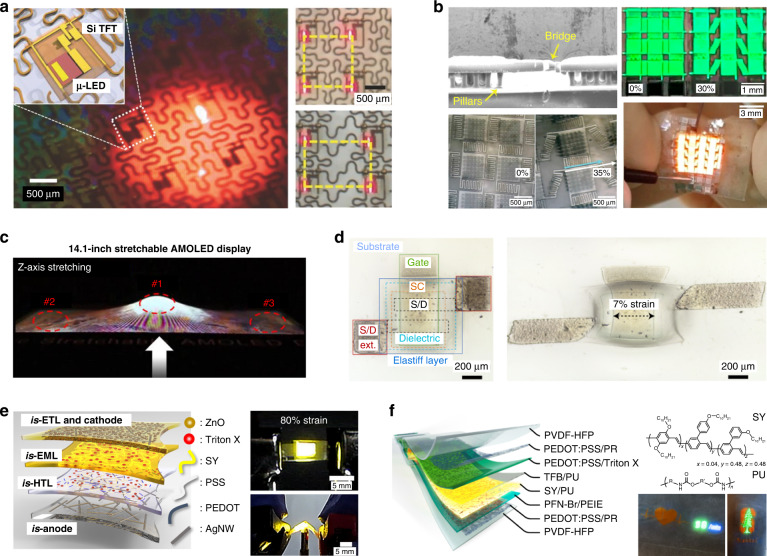
Basic technologies in the 3D free-form stretchable displays. **a** Serpentine-shaped interconnections and integrated µLEDs driven by Si-based TFTs. Reproduced with permission from ref. ^47^ Copyright 2017 Wiley-VCH. **b** Stretchable OLED display deposited on a patterned substrate with a stress-relief elastic pillar array and interconnection bridges. Reproduced with permission from ref. ^48^ Copyright 2020 American Chemical Society. **c** 14.1-inch stretchable active-matrix OLED display with wave-shaped interconnecting hinges. Reproduced with permission from ref. ^49^ Copyright 2019 Wiley-VCH. **d** Intrinsically stretchable transistor array integrated on a patterned elastomer layer with tunable stiffness. Reproduced with permission from ref. ^50^ Copyright 2021 Springer Nature. **e** Stretchable OLED composed of intrinsically stretchable (*is*-) layers. Reproduced with permission from ref. ^51^ Copyright 2021 AAAS. **f** Structure and light-emitting polymers of the on-skin stretchable all-polymer LED (APLED) displaying pulse signals in real-time. Reproduced with permission from ref. ^52^ Copyright 2022 Springer Nature

In the 2D expandable displays, deformation occurs only in specific parts of the display or along a fixed direction. The ultimate form factor innovation is realizing free-form displays that can be deformed in any position and along any direction of the display. This innovative deformability requests biaxial elastic characteristics without performance degradation. Approaches to this free-form displays can be largely divided into the structural design for minimizing the stress of rigid components in the stretched state^45–49^ and the material design for securing intrinsic stretchability of the device components^50–52^. The structural design has been widely investigated as a practical approach because the device components can be manufactured using the conventional production process^45–49^. The structural design has been primarily accomplished by creating relatively rigid island patterns on an elastic substrate and integrating the rigid device elements on the patterned regions^45^. Since the overall stress and strain are exerted selectively in the elastic substrate except the rigid patterns^46^, securing stretchable interconnections is essential for fabricating stretchable displays through the structural approach. Choi et al. applied the serpentine-shaped metal interconnections in fabrication of stretchable active-matrix µLED display driven by the single crystal Si-based TFTs (Fig. 6a)^47^. The µLEDs, Si-TFTs, and serpentine interconnections were transferred from the source wafers onto an elastomer substrate through a triple successive roll-transfer process. The µLED display was operated up to uniaxial strain (*ε*) = 40%, demonstrating the potential of manufacturing a practical stretchable display. Lim et al. devised the patterned upper substrate comprising the elastic pillars bonded to the bottom substrate and the bridges connecting the upper substrates. The elastic pillars decentralized the stress onto the devices and the bridges reduced the stress on the active area of devices under stretched state (Fig. 6b)^48^. OLEDs deposited on the upper substrate were operated stably under 30% biaxial strain. Samsung Display demonstrated a 14.1-inch stretchable active-matrix OLED display by using the wave-shaped hinge as an interconnection (Fig. 6c)^49^. The display panel showed successful operation during 10,000 stretching cycles at *ε* = 5%.

Material design for the free-form displays pursues intrinsic stretchability in all the device components, including the transistors (Fig. 6d) and light-emitting layers (Fig. 6e)^50–52^. All-polymer transistors and all-polymer light-emitting diodes would be an ideal platform for intrinsically stretchable displays. Wang et al. developed a strain-insensitive highly integrated (340 devices cm^−2^) stretchable transistor array using an all-elastomer strain engineering, where the patterned elastic layers with relatively high modulus were incorporated in the transistor area (Fig. 6d)^50^. All transistor components, including channels and driving electrodes, were composed of intrinsically stretchable materials. Kim et al. presented a stretchable all-polymer OLED, in which all the component layers (cathode, electron transport layer, emitting layer, hole transport layer, anode) were intrinsically stretchable (*is*-) (Fig. 6e)^51^. The display achieved 4400 cd m^−2^ at a low turn-on voltage (8 V) and maintained the luminescence under repeated cycles of 50% biaxial strain. Zhang et al. recently demonstrated a stretchable patterned all-polymer LED (APLED) colored yellow, red, green, and blue (Fig. 6f)^52^. The light-emitting polymer layers were composed of four different light-emitting polymers nanoconfined by phase separation in a polyurethane (PU) soft elastomer. Super yellow (SY), PPV copolymer, spiro-copolymer, and polyfluorene copolymer were used for yellow, red, green, and blue light-emitting polymers, respectively. These light-emitting layers were combined with the stretchable transparent polymer electrodes and interface-modified anode/cathode layers to realize the stretchable APLEDs. Through the optimization of material engineering and device fabrication, the APLED maintained high performance (7450 cd m^−2^) even at 100% strain and was able to display the pulse signals on the skin in real-time.

**Fig. 7:**
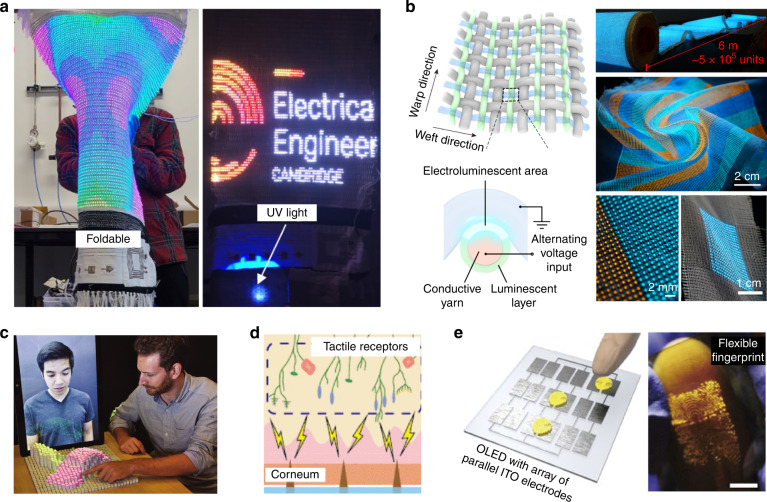
Crumpable and feelable displays. **a** Deformable smart textile display system with multifunctional devices. The right photograph shows the fiber photodetector that displays environmental UV light intensity. Reproduced with permission from ref. ^55^ Copyright 2022 Springer Nature. **b** Large-area ACEL textile display made by weaving conductive and luminescent fibers. Reproduced with permission from ref. ^56^ Copyright 2021 Springer Nature. **c** 3D shape-morphing system with interactive interfaces composed of mechanical actuators and visual-tracking systems. Reproduced with permission from ref. ^60^ Copyright 2015 IEEE. **d** Electric stimulation of the human skin mechanoreceptors. Reproduced with permission from ref. ^65^ Copyright 2013 IEEE. **e** AC-driven organic light-emitting board (OLEB) visualizing the finger touches on the OLEB. Reproduced with permission from ref. ^66^ Copyright 2017 Springer Nature

Since textiles are common substrates that can be crumpled in any direction, electronic textiles (e-textiles) have been spotlighted for highly flexible displays such as light-emitting fashionable clothes, digital signage, and curtain-type displays^53^. The initial monochromatic textile displays including a few luminescent fibers^54^ have evolved to a stand-alone full-color display fabricated by a large-scale weaving process^55,56^. Durability against mechanical washing in acidic/basic detergent conditions is critical in textile displays. Choi et al. developed a fully operational 46-inch smart textile display consisting of RGB fibrous LEDs (Fig. 7a)^55^. The textile display consisted of 120 × 65 × RGB LEDs (2.34 × 10^4^ subpixels) mounted on copper fibers. These LED fibers were asymmetrically woven with cotton fibers, so that they could be operated stably despite various mechanical deformations. The textile display was coupled with various fiber devices (radio frequency antenna, photodetector, touch sensor, temperature sensor, biosensor module, and energy storage) and visually displayed the information coming from the functional devices. For instance, the fiber photodetector sensed UV light and displayed the light intensity, which might be applied as a smart textile curtain for environmental monitoring. Alternating current electroluminescence (ACEL) lighting has a simple device structure, including two top-bottom electrodes with a phosphors-containing emitting layer^57^. The ACEL devices are mechanically durable because they require only spatial contact between the emitting layer and the electrodes, hence it is appropriate for large-scale textile displays^58^. Shi et al. weaved conductive weft fibers and luminescent warp fibers to form a 6 m-long, 25 cm-wide textile display (Fig. 7b)^56^. The display contained 5 × 10^5^ high-resolution electroluminescent units exhibiting nearly identical light intensity. Each electroluminescent unit could be programmed to control the emission by a driver circuitry. The display was crumpled and stretchable, and laundry was possible. The crumpable display showed potential as a smart wearable textile for the internet of things (IoT) and healthcare monitoring, however independent operation of the pixels driven by transistors has not been demonstrated so far.

Recent rise of augmented reality and metaverse platform request the future displays to combine vision with tactile sensation and to share physical information with others. The feelable displays synchronize the vision with tactility^59–61^. Leithinger et al. demonstrated a shape-morphing system, named inFORM, which provided real-time 3D surface shape change by using 900 linear vertical actuators (Fig. 7c)^60^. The motions of the hands were monitored by the camera and the actuators responded accordingly for synchronization. This work showed the potential capabilities of interactive operation and object manipulation by remote users. Using mechanical linear motors is not applicable to free-form displays^61^. Soft polymer-based piezoelectric actuators can work at low operation voltages and provide delicate control of the mechanical force, however, the mechanical power to be obtained is limited for expressing a broad range of tactility^62^. Miniaturized inorganic piezoelectric systems in a multiple-layer format can be a possible way to achieve both low operation voltage and large mechanical power^62^. Dielectric actuators which are based on ionic polarization can give deformability although their operating voltage is too high (>kV) for independent operation by transistors^63^. Tactile systems without requesting shape morphing have been also investigated. Electrovibration (EV) generates a vibrating electrostatic force when a finger is moving, hence it provides friction force vibration^64–66^. Although the feeling is obtained only when the finger moves and a high spatial resolution is difficult to achieve, EV can be applied immediately to the current displays as an indicator notifying simple information. Direct electrical stimulation of mechanoreceptors in the fingers has been investigated as a possible route to artificial tactility (Fig. 7d)^65^. Kim et al. successfully demonstrated an alternating current (AC)-driven organic light-emitting board (OLEB) as an interactive display visualizing external touch on the board by conductive objects of various shapes and materials (fingers, smart pens, metals, etc.) (Fig. 7e)^66^. To integrate the tactile system on a display, the tactile system should be invisible and standardization of stimulation and feeling should be achieved.

**Fig. 8:**
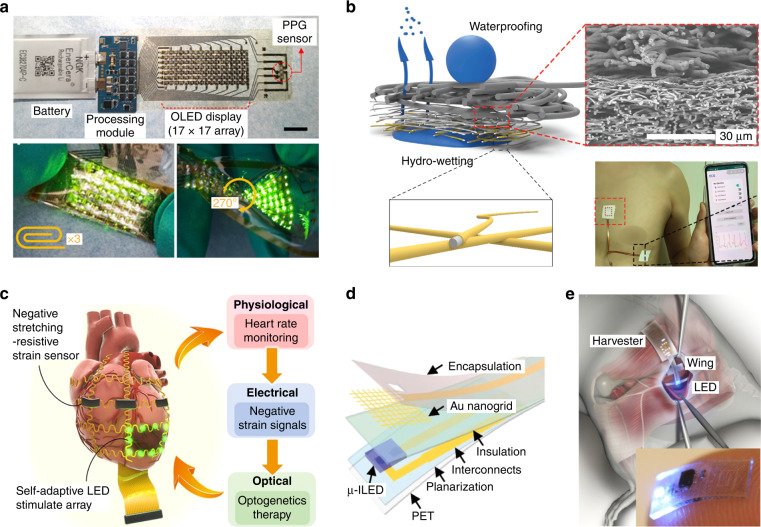
On-skin displays and implantable optoelectronic devices. **a** Stand-alone health monitoring patch containing a stretchable OLED array powered by an external battery. Reproduced with permission from ref. ^67^ Copyright 2021 AAAS. **b** On-skin electrocardiogram (ECG) sensor composed of permeable Au nanofiber mat with both waterproofing and hydro-wetting properties. Reproduced with permission from ref. ^69^ Copyright 2022 Wiley-VCH. **c** Cardiac optogenetics therapy composed of strain sensors for heart rate monitoring and self-adaptive LEDs for optical stimulation. Reproduced with permission from ref. ^74^ Copyright 2021 AAAS. **d**, **e** Use of implantable optoelectronic devices for wireless optical stimulation (**d**) and neuromodulation (**e**). Reproduced with permission from ref. ^75^ Copyright 2020 Wiley-VCH. Reproduced with permission from ref. ^76^ Copyright 2015 Springer Nature

Ultimate wearable displays are the systems-on-a-body such as the imperceptible on-skin displays and the implanted displays^67–70^. Because the on-skin displays require free-form deformability along with the skin layer, biaxial stretchability, and unconventional thinness are required. Practical utilization of the on-skin displays requires stable and long-term operation with permeability on the skin as well as integration with sensors, batteries or supercapacitors, and signal processors. Figure 8 shows the uses of the deformable displays as skin-attached and implantable devices, in addition to an example method to secure stable interconnects with waterproof and air/water permeability.

Samsung Electronics developed a standalone skin-like health monitoring patch that is composed of stretchable OLED displays, stretchable organic photoplethysmography (PPG) sensors, a flexible battery, and a processing module (Fig. 8a)^67^. The OLED components were mounted on the stress-relief layer of the stretchable substrate to minimize the stress applied to each pixel. The stretchable OLED display and the PPG sensor were conformally attached to the skin and operated without notable degradation under various mechanical deformations including crumpling, twisting, folding, and 30% stretching. To acquire long-term use of the on-skin displays, rapid permeability of air and water from the skin should be assured, while the surface of the display should be waterproofing for daily life uses in water environments. Nano- or microporous substrates with high-definition metal interconnections have been investigated to achieve air/water permeability^68–70^. Miyamoto et al. developed a stretchable Au nanomesh interconnection that can be conformally attached to the skin without a support substrate^68^. The nanomesh interconnection was fabricated by depositing Au on a poly(vinyl alcohol) (PVA) nanofiber mat followed by dissolving PVA in water. Oh et al. sputtered Au on the inner surface of a biocompatible styrene-ethylene-butylene-styrene (SEBS) elastomer nanofiber mat to create a stretchable interconnection (Fig. 8b)^69^. They utilized an imidized elastomer nanofiber mat to obtain strong binding of the Au shell to the nanofiber surfaces and maintained stable electrical conductivity under 50% biaxial stretching. The inner surface of the nanofiber mat was hydro-wetting, while the outer surface was waterproofing. The electrocardiogram (ECG) sensor made by the Au electrodes could successfully monitor the cardiac signals without being affected by various daily activities including exercising, showering, and sleeping. Ma et al. printed liquid metal onto an electrospun thermoplastic fiber mat to fabricate stretchable interconnections with good air permeability^70^. The breakup of liquid metal upon stretching and instantaneous oxide formation on the broken liquid metal led to the porous and buckled structure. The porous liquid metal layer on the fiber mat did not irritate the skin and the mounted LEDs were operated stably under large strain. So far, fabrication of on-skin displays with good gas/air permeability has not been demonstrated.

Implantable display with functional electronics is expected to go beyond the on-skin display. Since the implanted optoelectronic devices contain various device components (LEDs, antenna, signal processor, data transmission unit, energy storage unit, or biological sensors), electrochemical inertness and minimal invasive effects on living tissue are the key issues in addition to deformability^71–76^. Although implanted displays under the skin are the target for visional display, it has not been demonstrated yet. Research related to implantable display has focused mainly on integrating µLEDs for optogenetics therapy^72^ or neurostimulation^73^. Although the optogenetic therapy and optical neural stimulation studies do not include actual visual displays, the driving, and utilization of µLEDs in the body are closely related to the technological development of implanted deformable displays. Further advances in sophisticated implantable optogenetic devices are expected to be fundamental for implantable visualization. Hong et al. demonstrated an optogenetics therapy based on a closed-loop system consisting of a strain sensor array for heart rate monitoring, a processing circuit, and LEDs with self-adaptive light intensity control (Fig. 8c)^74^. The strain sensor array wrapping around the heart could detect negative resistance variation over a wide strain range. The signal processor raised an alarm once the heart rate exceeded the threshold of ventricular tachycardia (VT), then the optical regulation from the processor triggered self-adaptive light stimulation. Obaid et al. simplified the sensing and optogenetic stimulation by utilizing a transparent flexible Au nanogrid as a sensing electrode to minimize light artifacts during measurement (Fig. 8d)^75^. The device could record abnormal heart rhythms and restore sinus rhythm through optical pacing. Integrating transparent colocalized interfaces can be a versatile approach to minimizing optical disruption in implanted displays with biological functions. Park et al. developed a fully implantable optoelectronic device under the skin layer for wireless optogenetic modulation of the spinal cord and the peripheral nervous system (Fig. 8e)^76^. The entire device was designed to be soft in a thin elastomer film and reversibly activated the peripheral and spinal pain nerves of a freely moving mouse.

## Conclusions

Since the trends of pursuing personalization of electronic devices, the convenience of device holding, and preference for wide-screen display are expected to be accelerated further, commercialization of thin, light, and highly expandable/deformable displays will be of continuous industrial interest. Table 1 summarizes the required technological advances for the practical fabrication of the deformable displays and the technology readiness level (TRL) for each technology.

**Table 1:** Required technologies and assessments for the deformable displays

Technologies	Form factor	Requirements	Technology readiness level
Deformable hinge system	2D expandable	- Mechanical stability	On market
Screen crease prevention	2D expandable	- Minimum distortion of image quality (Color, resolution)	On market
Stress-regulated structure	2D expandable & 3D free-form	- Stretchability - Prevention of structural damage	Prototype
Stretchable transistors and light-emitting devices	3D free-form	- Electrical properties (ex. Mobility) - Optical performance (ex. Luminance) - High-density integration	Lab-scale
Stretchable interconnections & electrodes	3D free-form	- Metallic conductivity - Micro-, nanoscale resolution - High-density integration	Lab-scale
Stretchable anisotropic conductive film	3D free-form	- Electrical performance during deformation - High-density integration	Lab-scale
Stretchable passivation layers	3D free-form	- Passivation of water/oxygen - Stability under deformation	Lab-scale
Device integration on soft electrodes	3D free-form	- Mechanical stability - High-density integration	Lab-scale
Washable textile displays	3D free-form	- Water-proof - Mechanical stability during folding, curling	Lab-scale

The 2D expandable displays on market are based on conventional display panels so that their display performance and spatial resolution are not degraded. However, mechanical failure of the hinges due to repeated deformation for many years and deterioration of the display quality caused by the creases or wrinkles in the fold area of the screen still remain challenges to be solved. To realize the large-scale expansion in the industry, multi-axis folding, and paper-like thin film displays should be developed with new concepts to suppress the stress effect as well as remarkable advances in materials and fabrication processes.

To overcome the strain issue, advanced strain engineering of the multiple layers and the use of stretchable interconnections with metallic conductivity should be investigated thoroughly. Multiple neutral planes in the display panel are crucial for extreme strain engineering and minimized stress effect^35,36^. Insertion of a patterned stress-regulated structure or use of a compact hinge can cancel out or uniformly distribute the stress in the display panel^38,40–42^. Partial laser-cutting of a substrate facilitates multi-axis deformation without degradation of light-emitting performance^44^. However, regarding these strain-engineering approaches, it is pointed out as a potential problem that severe deformation may not be readily achieved because the rigid parts in the display limit the strain and cause fatigue cracks and delamination.

Commercialization of the stretchable 3D free-form displays is expected to be used initially for low-resolution products (such as fashion industry, health monitoring, and signage), and then evolve to high-resolution displays. In the initial stage of the 3D free-from display, the development of the corresponding useful contents and stretchable components is necessary rather than fulfilling the high performance of the display itself.

For the free-form displays, each component layer of transistors (source, drain, dielectric, and channel) and light-emitting diodes (cathode, anode, injection and transport layers, and emissive layer) should be developed not only to be soft and deformable, but having compatible performance to conventional thin film transistors and LEDs. Especially, the stretchable interconnection and electrode technologies still have not achieved the high spatial resolution equivalent to that of the conventional display, and their conductivities are far lower than the contemporary metal electrodes. Serpentine- or wavy-shaped metal electrodes on ultrathin substrates can be a promising route, but their low resolution and limited stretchability are obstacles to commercialization. Recently, metal deposition on an ultrathin amorphous carbon film (UACF) through a conventional mask presented crumpable high-resolution interconnections^77^. The UACF had no electrical tunneling barrier in the thickness direction, and it was conductive in the lateral direction within 1 μm but insulating when the distance was above 2 μm. This unique electrical property and the thinness (<10 nm) maintained the conductivity of the fine-patterned Au interconnection and drove µLEDs under repeated crumpling cycles. The UACF was further utilized to prepare stretchable sputtered metal interconnections^16^.

Along with the development of stretchable interconnections and display devices, there are additional components to be developed to prevent device failure: deformable adhesive anisotropic conductive films (ACF), stretchable passivation layer, reliable integration process of μLEDs on the soft electrodes, and washable textile electronics. Since the structure of the electrodes and interconnections becomes complicated in the thickness direction to achieve high stability under harsh mechanical chemical conditions, ACF itself should be able to be deformable without loss in electrical conduction. OLED is a strong candidate for stretchable displays. Unfortunately, there has been no successful report on the stretchable passivation layer. The non-conductive passivation layer is also important for implantable devices because the wireless communication module can be integrated into the microprocessor unit when a non-conductive soft water passivation layer is used instead of the currently used metallic passivation layer.

Integration of rigid device components on the soft deformable circuit lines is a challenging fabrication issue because current integration has been carried out on rigid substrates or flexible polymer substrates. Textile electronics is a promising deformable wearable displaying devices. The circuit lines, sensors, and displaying units fabricated by direct printing should be laundry-resistant. Water-resistant lamination on the top and bottom of each device unit so that all the circuitry is completely covered by a deformable passivation coating layer can be a possible direction. Although there are many hurdles to commercialization of the stretchable 3D free-form displays, increasing public needs and fast technological advances will push their industrialization in the near future.

## Supplementary information

Supplementary Information
