# Supplementary material for: Fabrication of practical deformable displays: advances and challenges
Source: Light Sci Appl. 2023 Mar 4;12:61. doi: 10.1038/s41377-023-01089-3 (PMC9984414; doi:10.1038/s41377-023-01089-3)
Supplement: Supplementary file 1 — Supplementary Information [file 41377_2023_1089_MOESM1_ESM.docx]

Supplementary Information for

**Fabrication of practical deformable displays: advances and challenges**

Dong Wook Kim^1,3†^, Seong Won Kim^2†^, Gyujeong Lee^2†^, Jangyeol Yoon^2^, Sangwoo Kim^2^, Jong-Ho Hong^2^, Sung-Chan Jo^2^* and Unyong Jeong^1^*

Prof. U. Jeong, Dr. D. W. Kim

^1^Department of Materials Science and Engineering, Pohang University of Science and Technology (POSTECH), 77 Cheongam-ro, Nam-gu, 37673, Pohang, Gyeongbuk, Republic of Korea

^3^Physical Intelligence Department, Max Planck Institute for Intelligent Systems, Hisenbergstr. 3, 70569, Stuttgart, Germany.

Dr. S. Jo, Dr. S. W. Kim, Dr. G. Lee, Dr. J. Yoon, Dr. S. Kim, Dr. J. Hong

^2^Advanced Research Team, Samsung Display Corporation, 1 Samsung-ro, Yongin-si, Gyeonggi-do, Republic of Korea

^†^ These authors contributed equally

*Corresponding authors: [ujeong@postech.ac.kr](mailto:ujeong@postech.ac.kr), [enigma.jo@samsung.com](mailto:enigma.jo@samsung.com)

| **Technologies** | **Display type** | **Screening operators** |
| --- | --- | --- |
|  |  |  |
| 2D Expandable Displays | Dynamic (Foldable, Rollable, Slidable, Multi-Foldable) | ((Fold* or Bend* or Slid* or Roll*) or (Multi ADJ (Fold* or Bend* or Slid* or Roll*))) NEAR3 (Display or OLED or "LED" or (Light adj Emitting) or (Mobile adj Phone))  not Multi-axis |
|  | Paper-Like (Thin, Multi-Foldable) | (Thin or Slim or Paper or Flimsy or Wrinkle-free or Crease-free ((Multi-axis or Omni) ADJ (Fold* or Bend* or Slid* or Roll*)))  NEAR3 (Display or OLED or "LED" or (Light adj Emitting) or (Mobile adj Phone)) not (Multi ADJ (Fold* or Bend* or Slid* or Roll*)) not (thin ADJ film) |
| 3D Free-Form Displays | Crumpable | (Textile or Fabric or Woven or Fiber or Crump* or Grasp*) NEAR3 (Display or OLED or "LED" or (Light adj Emitting) or (Mobile adj Phone)) not (optical ADJ fiber) |
|  | On-Skin | (Stretch* or On-skin or Skin-like or Epidermal or ((Body or Skin) ADJ3 (attach* or wear*)))  NEAR3 (Display or OLED or "LED" or (Light adj Emitting) or (Mobile adj Phone)) |
|  | Feelable | (((Tactile or Texture or Stimul*) NEAR3 (Feel* or Sens* or Respons* or Detect*)) or Opto-tactile or Mechano-Chromic or Mechanochromic  or Piezo-Chromic or Piezochromic or Haptic or (Haptic ADJ Feedback)) NEAR3 (Display or OLED or "LED" or (Light adj Emitting) or (Mobile adj Phone)) |
|  | Implantable | ((Body or Organ or Bio) ADJ3 (Implant* or Compatib* or Degrad* or Integrat*))  NEAR3 (Display or OLED or "LED" or (Light adj Emitting) or (Mobile adj Phone)))  not Drug |

**Table S1**. Screening operators for the patents shown in Fig. 2
